# Errors From Asymmetric Emission Rate in Spaceborne, Limb Sounding Doppler Interferometry: A Correction Algorithm With Application to ICON/MIGHTI

**DOI:** 10.1029/2020EA001164

**Published:** 2020-10-07

**Authors:** Yen‐Jung J. Wu, Brian J. Harding, Colin C. Triplett, Jonathan J. Makela, Kenneth D. Marr, Christoph R. Englert, John M. Harlander, Thomas J. Immel

**Affiliations:** ^1^ Space Sciences Laboratory University of California Berkeley CA USA; ^2^ Department of Electrical and Computer Engineering University of Illinois at Urbana‐Champaign Urbana IL USA; ^3^ Space Science Division U.S. Naval Research Laboratory Washington DC USA; ^4^ Space Systems Research Corporation Alexandria VA USA

**Keywords:** Dopper interferometry, asymmetric ionosphere, neutral wind retrieval, Ionospheric Connection Explorer

## Abstract

The Michelson Interferometer for Global High‐resolution Thermospheric Imaging (MIGHTI) on NASA's Ionospheric Connection Explorer (ICON) mission is designed to measure the neutral wind and temperature between 90 and ∼300 km altitude. Using the Doppler Asymmetric Spatial Heterodyne (DASH) spectroscopy technique, observations from MIGHTI can be used to derive thermospheric winds by measuring Doppler shifts of the atomic oxygen red line (630.0 nm) and green line (557.7 nm). Harding et al. (2017, https://doi.org/10.1007/s11214‐017‐0359‐3) (Harding17) describe the wind retrieval algorithm in detail and point out the large uncertainties that result near the solar terminators and equatorial arcs, regions of large spatial gradients in airglow volume emission rates (VER). The uncertainties originate from the assumption of a constant VER at every given altitude, resulting in errors where the assumption is not valid when limb sounders, such as MIGHTI, observe regions with significant VER gradients. In this work, we introduce a new wind retrieval algorithm (Wu20) with the ability to account for VER that is asymmetric along the line of sight with respect to the tangent point. Using the predicted ICON orbit and simulated global VER variation, the greatest impact of the symmetric airglow assumption to the ICON vector wind product is found within 30° from the terminator when the spacecraft is in the dayside, causing an error of at least 10 m/s. The new algorithm developed in this study reduces the error near the terminator by a factor of 10. Although Wu20 improves the accuracy of the retrievals, it loses precision by 75% compared to Harding17.

## Key Points

- An algorithm is introduced that improves the accuracy of limb‐sounding wind retrievals, accounting for the asymmetric volume emission rate
- The algorithm is tested with simulated measurements of the ICON/MIGHTI instrument and shown to significantly improve retrieval's accuracy
- The algorithm improves accuracy but at the expense of precision in regions of large asymmetry

## 1 Introduction

NASA's Ionospheric Connection Explorer (ICON) (Immel et al., 2018) is a mission targeted on the dynamics of the upper atmosphere in order to quantify forcing from below and above this region. The Michelson Interferometer for Global High‐resolution Thermospheric Imaging (MIGHTI) is one of the instruments onboard ICON. MIGHTI is designed to measure the neutral winds and temperatures in the mesosphere and thermosphere. Using the Doppler Asymmetric Spatial heterodyne (DASH) spectroscopy technique (Englert et al., 2007, 2017), observations from MIGHTI can be used to derive neutral winds by measuring Doppler shifts in the observed fringe patterns of the atomic oxygen red line (630.0 nm) and green line (557.7 nm). Here we focus on neutral wind retrievals only, since the neutral temperature is derived from a different method (Stevens et al., 2018). This Doppler shift measurement enables the retrieval of the wind velocity in the altitude range of 90 to ∼300 km (Harlander et al., 2017). MIGHTI includes two identical instruments oriented such that the lines of sight (LOSs) are perpendicular to each other, with MIGHTI‐A and MIGHTI‐B's LOS at an azimuthal angle of ∼45° and ∼135°, respectively, relative to the spacecraft velocity vector. This setup allows MIGHTI to measure the horizontal LOS wind components in two orthogonal directions and then reconstruct the meridional and zonal winds at all covered altitude levels.

The fundamental physics behind the measurements made by MIGHTI is the Doppler effect, according to which the frequency of the observed light changes when the target is moving with a velocity component along the LOS. The MIGHTI instrument generates an interferogram, whose phase shift is directly related to the Doppler velocity of the photons emitted along the LOS, and can therefore be analyzed to retrieve the wind velocity. As with most Abel‐like inversions, wind retrieval algorithms based on limb sounding Doppler shift interferometry assume that atmospheric parameters vary only in altitude, not in latitude nor longitude. However, for the case of retrieving winds from LOS‐integrated observations of volume emission rate (VER), this assumption is often violated due to the asymmetric distribution of VER, which also referred to as a “locally spherically inhomogeneous distribution of VER” in other studies.

Using National Center for Atmospheric Research Thermosphere‐Ionosphere‐Electrodynamics General Circulation Model (NCAR/TIEGCM) (Richmond et al., 1992), Harding et al. (2017) showed that large errors in the wind retrievals occur when either of MIGHTI's LOS cross the solar terminator or the edge of the equatorial ionization anomaly (EIA). For the sake of convenience, “terminator” mentioned in this paper refers to the solar terminator. The error in those particular areas can be larger than 10 m/s for both red and green emission lines, which is significant relative to the magnitude of planetary waves and tides in the thermosphere (Hagan & Forbes, 2003). Additionally, ionospheric irregularities that have a size larger than 500 km can potentially negatively impact the accuracy of wind retrievals, since the along‐track resolution of MIGHTI is approximately 500 km (Englert et al., 2017).

Experience from the Wind Imaging Interferometer (WINDII) on the Upper Atmosphere Research Satellite (UARS) (Shepherd et al., 1993) and from the Doppler Interferometer (TIDI) on the Thermosphere, Ionosphere, Mesosphere Energetics, and Dynamics (TIMED) spacecraft (Killeen et al., 2006) has also shown that wind velocity retrievals near the terminators are less reliable, because of the breakdown of the assumption of VER symmetry along the LOS. The day‐night transition at the terminators drives important interactions between neutral winds and plasma and plays a crucial role in the *E* and *F* region ionosphere dynamics (Somsikov, 2011). This interaction leads to phenomena such as solar terminator waves (Liu et al., 2009), prereversal enhancement (PRE) of the upward ionospheric plasma drifts (**E** ×**B**) (Chen et al., 2017; Heelis et al., 2012; Kelley et al., 2014; Verhulst & Stankov, 2017), plasma irregularities (Makela, 2006; Otsuka, 2018), large‐scale traveling ionospheric disturbances (LSTIDs) (Song et al., 2013), and medium‐scale traveling ionospheric disturbances (MSTIDs) (Hernández‐Pajares et al., 2006). ICON's low‐inclination orbit of 27° provides 29 terminator crossings per day allowing ICON to monitor the zonal transition of the *E* and *F* region neutral winds near the terminator. Therefore, a data analysis algorithm with an improved performance under the conditions experienced near the terminator would yield to useful scientific results.

Harding et al. (2017), hereafter referred to as Harding17, describe a wind retrieval algorithm under the assumption of a locally spherically symmetric airglow VER and wind. Based on Harding17, we propose a new improved algorithm, which keeps the symmetric wind assumption but allows considering asymmetric VER case, and, for simplicity, we refer to it as Wu20 in this study. Wu20 takes into account the VER difference before and after the tangent point to reduce the effect of the asymmetry of the airglow VER on wind retrievals from ICON or any other Doppler limb sounder. The theory of the LOS wind retrieval using the symmetric airglow assumption in the onion peeling method in Harding17 is given in section 2.1 of this study. Our newly developed algorithm, Wu20, which is able to accommodate a significant brightness gradient structure in the wind retrieval process is described in section 2.2. The idea of considering asymmetric airglow has not been implemented in previous spaceborne Dopper shift instruments since it is difficult to know the VER gradient in advance. However, a simplified method, with the assumption that the VER ratio variation as a function of longitude is the same for all altitudes, is demonstrated in section 3 for the spaceborne measurement in the low‐inclination angle orbit like ICON. The trade‐off between the accuracy and precision of the new algorithm is discussed in section 4. Further remarks and conclusions are given in the last section.

## 2 Theory and Methodology

The DASH spectroscopy technique is partially inherited from the Stepped Michelson Interferometer (SMI) (Shepherd et al., 1993). Similar to SMI, DASH uses the fact that a small frequency shift of a single emission line results in a phase shift in the interferogram that increases with increasing optical path difference (OPD) (Englert et al., 2007; Harlander et al., 2017). Similar to conventional Spatial Heterodyne Spectrometer (SHS) (Harlander et al., 1992), DASH has fixed and tilted gratings, so that the OPD across the aperture of the interferometer is imaged onto the detector (Englert et al., 2017; Harlander et al., 2017). Therefore, one can make use of all elements of the detector array to reconstruct the phase shift in a larger number of OPD samples than the traditional SMI. The phase shift, Δ*ϕ*, of a single emission line as a function of the OPD is given by (Englert et al., 2007)

(1) $$\Delta \phi =\frac{2\pi d}{\lambda c}v,$$

where Δ*ϕ* is the Doppler phase shift; *λ* is the non‐Doppler‐shifted wavelength, which is assumed to be 630.0 nm for the red line and 557.7 nm for the green line; *c* is the speed of light; *v* is the Doppler velocity along the LOS; and *d* is the OPD, which is a known parameter determined by the design parameters of the instruments. Therefore, Doppler phase shift is the only unknown parameter in Equation 1 needed in order to derive the Doppler velocity. Subsections 2.1 and 2.2, describe the algorithm to retrieve the Doppler phase shift Δ*ϕ* using the symmetric airglow assumption and our newly proposed asymmetric airglow procedure, respectively.

### 2.1 LOS Wind Retrieval Using the Symmetric Airglow Assumption in Harding17

In section 2.1, we summarize the inversion algorithm used for wind retrieval described by Harding17. According to Harding17, in a calibrated interferogram, the zero‐wind phase and constant background are removed from the original LOS‐integrated interferogram. The calibrated interferogram is a two‐dimensional array in complex number format for which the horizontal and vertical dimensions are the OPD and the tangent altitude levels, respectively. The complex number array contains information on the amplitude of the integrated LOS brightness and the Doppler phase shift measured by each OPD. A single row of the calibrated interferogram is denoted *I*(*x*):

(2) $$I(x)=\int_{0}^{\infty }B^{obs}(k)e^{j(2\pi kx)}dk,$$

where *B*^*obs*^(*k*) is the observed spectral intensity and *k* is proportional to the heterodyned spatial fringe frequency of the interferogram. *j* is the unit imaginary number. *x* is the position along the horizontal axis of the detector, which is related to the OPD.

The spectrum observed by the field of view (FOV) of the *m*th row of a charge‐coupled device (CCD) is denoted $B_{m}^{obs}(k)$, which is the integration of the contributions from all sections along the LOS, *ds*. The size of the pixel is the thickness of the layer that an instrument can resolve; therefore, the spectral intensity that varies within one pixel is ignored since it is below the instrumental resolution limit. The Doppler shift, Δ*k*, is induced by the altitude‐dependent wind along the LOS:

(3) $$B_{m}^{obs}(k)=\int_{0}^{\infty }B(k-\Delta k,h)ds.$$

As discussed in Harding17, we use *m* and *n* for the LOS and the altitude layer, respectively. The highest pointing LOS and the corresponding top altitude layer are represented by *m* = 0 and *n* = 0, respectively. In order to estimate all *B*(*k* − Δ*k*, *h*) along the LOS, similar to Harding17, we assume the airglow VER and wind velocity are independent of latitude and longitude, but constant over small altitude layers. With this assumption, Equation 3 becomes

(4) $$B_{m}^{obs}(k)=\overset{N-1}{\underset{n=0}{\sum }}B_{n}(k-\Delta k_{mn})w_{mn},$$

where Δ*k*_*mn*_ is the Doppler shift induced by the LOS component of the wind at the *n*th layer for the *m*th LOS and *w*_*mn*_ is the pathlength of the *n*th layer in the *m*th LOS.

Replacing *B*(*k*) in Equation 2 with $B_{m}^{obs}(k)$ in Equation 4, the intensity of the calibrated interferogram for the *m*th LOS, *H*_*m*_(*x*), becomes

(5) $$H_{m}(x)=\overset{N-1}{\underset{n=0}{\sum }}\left(\int_{0}^{\infty }B_{n}(k-\Delta k_{mn})e^{j(2\pi kx)}dk\right)w_{mn}.$$

Equation 6 below is written in the form of *k*, with the so called uniform wind assumption: Harding17 assumes the Doppler shift of the wind is the same throughout each altitude layer, so that the wave number‐related term, *k*, can be replaced by *ϕ*_*n*_ and *M* is the total number of observation angles. Additionally, Δ*k*_*mn*_ in *B*_*n*_(*k* − Δ*k*_*mn*_) can be disentangled as $B_{n}(k)e^{j\Delta \phi_{n}cos\alpha_{mn}}$, where Δ*ϕ*_*n*_ is the phase shift in the *n*th layer and *α*_*mn*_ is the angle between the *m*th LOS and the tangential direction of the *n*th layer at their intersection. At the end, we combine the spectrum *B*_*n*_ and the phase shift term to derive the complex intensity of the interferogram of the *n*th layer *I*_*n*_.

(6) $$\begin{array}{ccc} H_{m}(x) & =\overset{M}{\underset{n=0}{\sum }}(B_{n}e^{j\Delta \phi_{n}cos\alpha_{mn}}e^{j\Delta \phi_{n}})w_{mn}\;\forall m\in [1,M-1] & \\ & =\overset{M}{\underset{n=0}{\sum }}I_{n}e^{j\Delta \phi_{n}cos\alpha_{mn}}w_{mn}\;\forall m\in [1,M-1]. \end{array}$$

The angle between the zeroth LOS and tangent of the zeroth layer *cosα*_00_ = 1 at the top layer, so that the relation between the brightness and phase shift described in Equation 6 becomes

(7) $$I_{0}(x)e^{j\Delta \phi_{m}}=\frac{1}{w_{00}}H_{0}(x),$$

where *H*_0_(*x*) is the single‐row brightness of the top layer and *w*_00_ is the distance of the top LOS passing through the top layer. This is the first step of the so called onion peeling method.

The major steps in the onion peeling method are summarized below: (1) using the observed intensity of the tangent layer of the top LOS to estimate the averaged brightness of the same spherical layer; (2) estimating the pathlength of each layer on the same LOS under the spherical Earth assumption; and (3) with 1 and 2, once we know the brightness of the top layer, the brightness of the layer below can be obtained by removing (“peeling”) the brightness of the top layer from the intensity of that LOS.

In order to perform the onion peeling method on the lower layers, Equation 7 can be iteratively extended down to other layers, in which the intensity of the *n*th layer in the *m*th LOS is given as

(8) $$I_{m}(x)e^{j\Delta \phi_{m}}=\frac{1}{w_{mm}}\left(H_{m}(x)-\overset{m-1}{\underset{n=0}{\sum }}I_{n}(x)e^{j\phi_{n}cos\alpha_{mn}}w_{mn}\right)\;\forall m\in [1,M-1],$$

where *I*_*m*_(*x*) is the brightness of the tangent height of the *m*th LOS and *I*_*n*_(*x*) is the brightness of the *n*th layer that needs to be removed.

With the assumption of a spherically symmetric airglow, *w*_*mn*_ is simply the pathlength of the *m*th LOS through the *n*th layer:

(9) $$w_{mn}=2\left(\sqrt{r_{n+1}^{2}-r_{m}^{2}}-\sqrt{r_{n}^{2}-r_{m}^{2}}\right),$$

where *r*_*n* + 1_ and *r*_*n*_ are radial distances of the middle point of *n*th and (*n* + 1)th layer from the center of the earth and *r*_*m*_ is the radial distance of the tangent point of the *m*th LOS.

**Figure 1:**
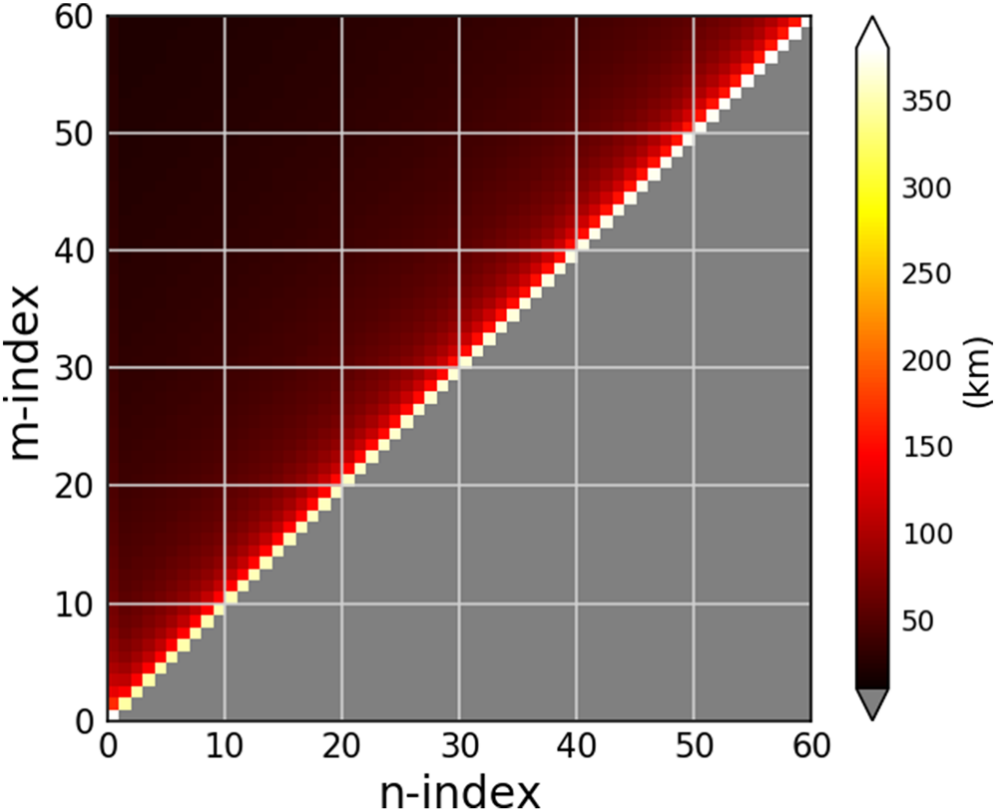
The pathlength matrix used in the symmetric airglow assumption for the red line observation on ICON/MIGHTI. The *m* index is for the *m*th LOS, and the *n* index is for the *n*th altitude layer. The gray area (representing 0 values) is for the invalid cases where *m* < *n*. The diagonal elements of the matrix are for the case when the LOS goes through the tangent layer, *m* = *n*. The path length of *m* = *n* case is significantly longer than the *m* ≠ *n* cases. For instance, *w*_*mn*_ = 348 km when *m* = *n* = 30, while *w*_*mn*_ = 150 km when *m* = 30 and *n* = 29.

The pathlength matrix *w* is defined by the orbit of the spacecraft, the resolution of the vertical sampling, and the pointing of the LOS. The visualization of *w* is demonstrated in Figure 1, in which we use the red line observations on ICON/MIGHTI as an example, with 2.5‐km vertical sampling size viewing the Earth's limb from a circular orbit and the altitude of ∼575 km. The gray area represents the invalid cases where the altitude of the *n*th layer is lower than the tangent point of the *m*th LOS, *m* < *n*. The diagonal elements of the matrix are for the case when the LOS goes through the tangent layer, *m* = *n*. The path length of *m* = *n* case is significantly longer than the *m* ≠ *n* cases. For instance, *w*_*mn*_ = 348 km when *m* = *n* = 30, while *w*_*mn*_ = 150 km when *m* = 30 and *n* = 29.

### 2.2 LOS Wind Retrieval Using the Asymmetric Airglow Procedure in Wu20

Harding17 pointed out that an asymmetric airglow can have an impact on the accuracy of wind retrievals. In section 2.2, we will introduce an extended algorithm, Wu20, which is applicable in the asymmetric VER case. With inheriting the onion peeling method, Wu20 considers the VER difference before and after the tangent point. Unlike the symmetric airglow case, it is not valid in an asymmetric airglow procedure to use the full distance (Equation 9) of the LOS passing through the target layer to calculate the contribution from each layer. Taking the brightness variation along the LOS into account, the onion peeling method described in Equation 8 can be modified for the asymmetric VER procedure:

(10) $$I_{m}(x)e^{j\Delta \phi_{m}}=\frac{1}{w_{mm}}H_{m}(x)-\frac{1}{w_{mm}}\left(\overset{m-1}{\underset{n=0}{\sum }}e^{j\phi_{n}cos\alpha_{mn}}(I_{mn,b}(x)w_{mn,b}+I_{mn,a}(x)w_{mn,a})\right).$$

Following the denoting convention in the symmetric VER case in Harding17, *I*_*mn*,*a*_ and *I*_*mn*,*b*_ are the brightnesses in the *n*th layer of the *m*th LOS after and before the tangent point, while *w*_*mn*,*a*_ and *w*_*mn*,*b*_ are the *n*th layer lengths of the *m*th LOS after and before the tangent point. Considering the VER contribution before and after the tangent point, we can adjust the brightness difference along the LOS in the onion peeling method.

**Figure 2:**
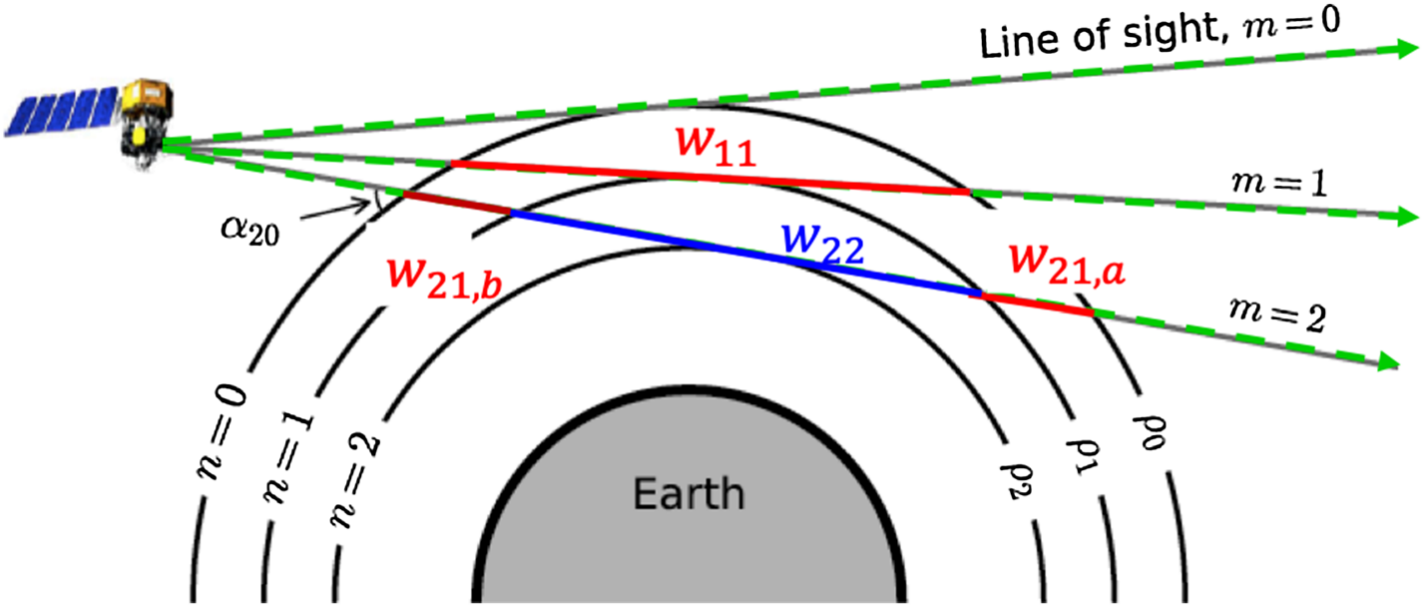
Illustration showing the relative geometry of pathlengths (*w*_*mn*,*a*_, *w*_*mn*,*b*_, and *w*_*cc*_) under the assumption of an asymmetric airglow. The pathlengths *w*_21,*a*_ and *w*_21,*b*_ are the length of the (*n* = 1)th layer in the (*m* = 2)th LOS after and before the tangent point, respectively. When *n* = *m*, *w*_11_, for example, it is the pathlength at the tangent layer of the LOS. The figure is a modified version of the Figure 2 in Harding et al. (2017)

In addition to *w*_*mn*,*a*_ and *w*_*mn*,*b*_ described above, a special case is when *m* = *n* = *c*, *w*_*cc*_ is the pathlength of the tangent layer of the *c*th and *c*th LOS. The corresponding brightness of *w*_*cc*_ is denoted as *I*_*cc*_. In the symmetric VER case (8), we use *I*_*n*_ only to represent the brightness of the *n*th layer since the brightness is constant throughout the same altitude (*I*_*n*_ = *I*_*mn*,*a*_ = *I*_*mn*,*b*_ = *I*_*nn*_). In the asymmetric VER procedure, on the other hand, *I*_*mn*,*a*_, *I*_*mn*,*b*_, and *I*_*nn*_ can be different. Figure 2 shows the relative geometry of the pathlengths that are used in the asymmetric VER procedure. The pathlengths *w*_21,*a*_ and *w*_21,*b*_ are the length of the (*n* = 1)th layer in the (*m* = 2)th LOS after and before the tangent point, respectively. *w*_22_ is the pathlength at the tangent layer of the (*m* = 2)th LOS.

Recall that the left‐hand side of Equation 10 is the brightness of the tangent altitude. Now we introduce *R*_*mn*,*ξ*_ (*R*_*mn*,*ξ*_ ∈ {*R*_*mn*,*a*_,*R*_*mn*,*b*_}), which is the ratio of the brightness at *w*_*mn*,*ξ*_ (*w*_*mn*,*ξ*_ ∈ {*w*_*mn*,*a*_,*w*_*mn*,*b*_}) with respect to the brightness at *w*_*nn*_. *R*_*mn*,*ξ*_ allows performing the asymmetric VER procedure without knowing the absolute brightness:

(11) $$R_{mn,\xi }=\frac{I_{mn,\xi }}{I_{nn}}\;\xi =a,b.$$

Under the spherical Earth assumption, which is also applied in Harding17, *w*_*mn*,*ξ*_ = *w*_*mn*_/2, Equation 10 can be written as a function of *R*_*mn*,*ξ*_:

(12) $$I_{m}(x)e^{j\Delta \phi_{m}}=\frac{1}{w_{mm}}H_{m}(x)-\frac{1}{w_{mm}}\left(\overset{m-1}{\underset{n=0}{\sum }}I_{nn}(x)e^{j\phi_{n}cos\alpha_{mn}}\frac{w_{mn}}{2}(R_{mn,b}+R_{mn,a}\right)).$$

As expected, when the brightness is the same everywhere in the layer of the same altitude, *R*_*mn*,*b*_ = *R*_*mn*,*a*_ = 1, and Equation 12 reverts back to the symmetric airglow assumption case described in Equation 8.

**Figure 3:**
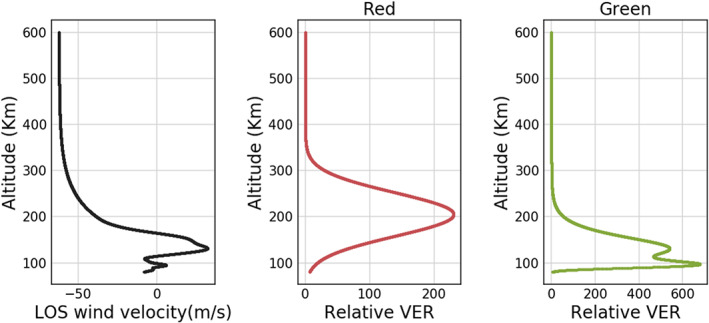
The input LOS wind velocity and VER of red and green lines used for creating the synthetic MIGHTI interferogram used in this study. The LOS wind and red line VER profile are the input for the model comparison shown in Figures 4, 6, and 8, whereas the LOS wind and green line VER profile are the input for the comparison shown in Figure 7.

In order to verify the validity of Equation 12, we perform a control run (Harding17) and a test run (Wu20) using the simulated interferogram for the thermospheric red line. We use ICON/MIGHTI's FOV and orbit to create the simulated interferogram *H*_*m*_(*x*), with a VER structure in the horizontal direction that exponentially falls off at the day‐to‐night terminator with a scale distance of 2,000 km, to mimic the measurement of the large VER gradient structure. The input VER profile and LOS wind profile used in this study are shown in Figure 3. Note that no other error sources are included in this example, such as noise from the instrument itself. Since both Harding17 and Wu20 rely on the onion peeling method (Harding et al., 2017), no noise‐reduction constraint is applied in the onion peeling method.

**Figure 4:**
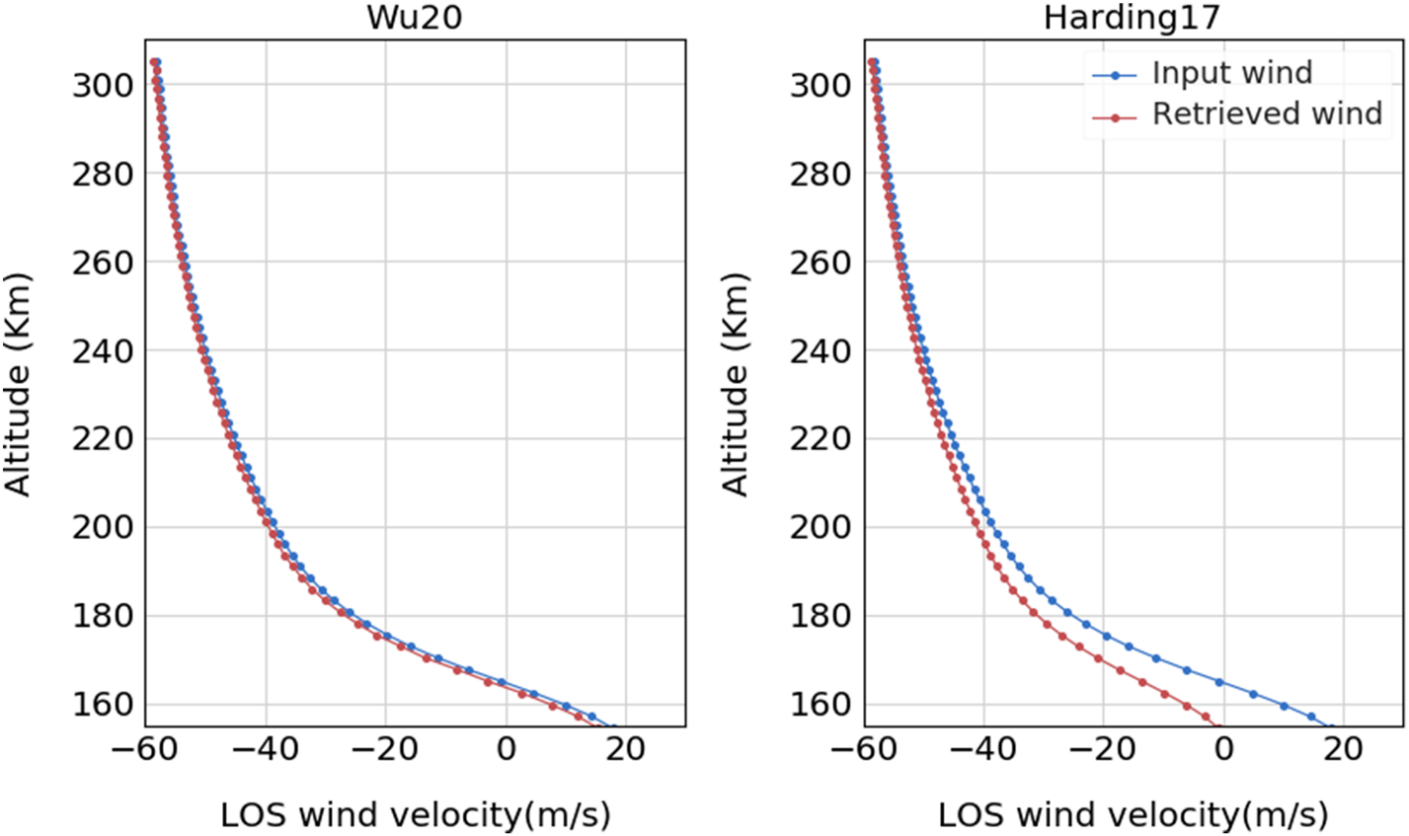
The comparison of the input wind (blue) and the retrieved wind (red) from the red line emission. The test run (left) uses the asymmetric VER assumption while the control run (right) adopts the symmetric VER procedure.

The control run uses *w*_*mn*_ with the symmetric VER assumption in Harding17, whereas the test run utilizes the Wu20 algorithm described above. Figure 4 shows the retrieved wind from the red line using the algorithm described in Wu20 (left) and Harding17 (right). Comparing the value of the input wind (blue) and retrieved wind (red), at the bottom altitude where the error is the largest, the difference is 0.8 m/s in the Wu20 run while it is 20.0 m/s in the Harding17 run, which confirms that the error of the retrieved wind can be minimized if we know the VER variation along the LOS. Of course, the VER variation is not strictly known in practice. In the next section, we describe an approximation that can be implemented for the case of the ICON viewing geometry.

## 3 Examining the Applicability of Wu20 in Spaceborne, Limb Sounding Doppler Interferometry: Using ICON/MIGHTI Observations as an Example

The previous section described an improved algorithm that can be used when the spatial distribution of VER is known a priori. Given the lack of a tomographic viewing architecture, the VER variation along the LOS is generally not available for Doppler shift wind retrieval instruments with which the LOS measurements are made. However, continuous brightness observations by ICON in a low inclination angle orbit allow us to work toward a similar capability. In this section, we take the actual ICON orbit into account and use simulated ICON/MIGHTI data as an example to examine the applicability and effectiveness of Wu20.

**Figure 5:**
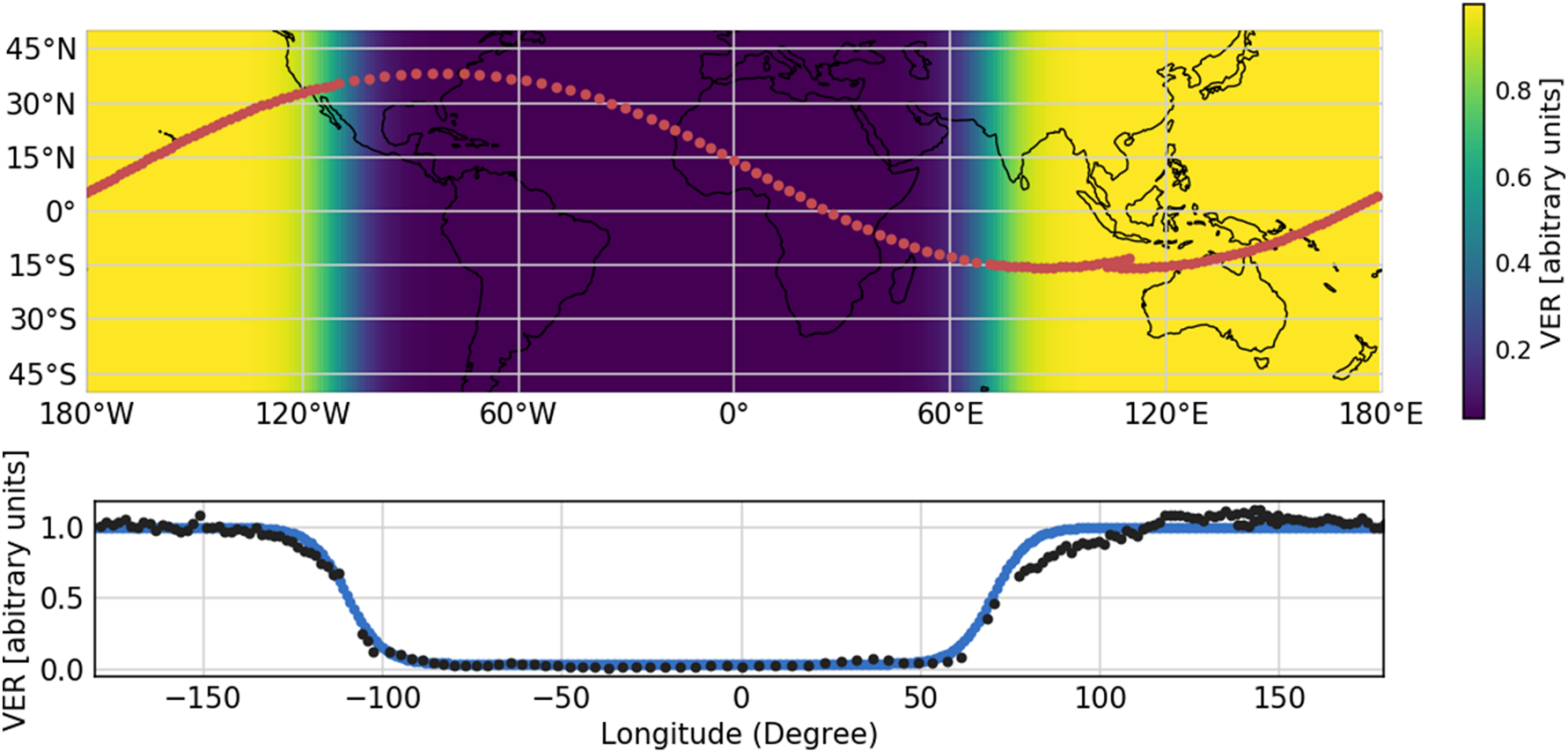
(Top) The geographic variation of VER used in the model. The VER depends on longitude only. The VER variation is applied in all altitudes and for both red and green lines. The red dots in the top plot show the ICON orbit also indicating the sampling cadence change of MIGHTI from 30 s in the daytime to 60 s in the nighttime. The orbit inclination is 27°. (Bottom) The black dots are the actual relative VER observed by ICON. The blue curve is a combination of two logistic functions representing the relative VER used in this simulation, which is fitted to the actual ICON measurements.

The predicted ICON location and MIGHTI LOS are used to create a synthetic interferogram by integrating the simulated VER along the LOS. Figure 5 (top panel) shows an example ICON orbit with a 27° inclination angle (red dots), with the actual sampling rate of MIGHTI, which is 30 s in the daytime and 60 s in the nighttime. To simulate the terminator transition that ICON encounters in every orbit, the day‐to‐night and night‐to‐day terminators are assumed to be at −110° and 70° longitudes, respectively. The shapes of the relative VER profiles for the green and red lines used in the simulation are taken from Figure 3 and do not change with latitude nor longitude while their absolute magnitudes are scaled according to the given VER variation in Figure 5. This resembles the case at the equinox when the subsolar point is on the equator. Also note that no other error sources are added in the simulation.

For the purposes of demonstrating the algorithm, the nominal terminator shape in Figure 5 is determined by fitting logistic functions to the relative VER observed by MIGHTI (black dots in Figure 5). This relative VER variation as a function of longitude is calculated from a single ICON orbit and taken from an observing altitude of 220 km, which is the brightest layer at the nighttime on 1 January 2020. This is during the solar minimum at the end of Solar Cycle 24, with a daily F10.7 of 69.2 solar flux units. The maximum VER of the fitted logistic function is 135 times larger than the minimum VER in this case. In order to focus on the large VER gradient at the terminators, this fitting aims at the decay and growth rates of the VER at the terminators only and ignores the variation of VER over the dayside or nightside. One more important assumption in the simulation is that the assumed wind vector is still spherically symmetric in the same altitude layer, whereas in reality, the wind is expected to be nonuniform, but this simplification will help in isolating the effect of an asymmetric VER.

Harding17 assumes that the VER and wind in the atmosphere do not change significantly in 5 to 8 min, which is an assumption that is used to combine two MIGHTI LOS wind components to obtain a cardinal wind vector. Wu20 uses the same assumption to characterize the asymmetries in the VER, which are needed for the LOS wind retrieval as described in Equation 12. These assumptions are not necessarily valid, but they are approximations that work well in practice, as we show in the following simulation. To obtain *R*_*mn*,*b*_ and *R*_*mn*,*a*_ in Equation 11, we generate a local VER ratio as a function of longitude, similar to Figure 5, from the time‐dependent intensity of each measurement.

**Figure 6:**
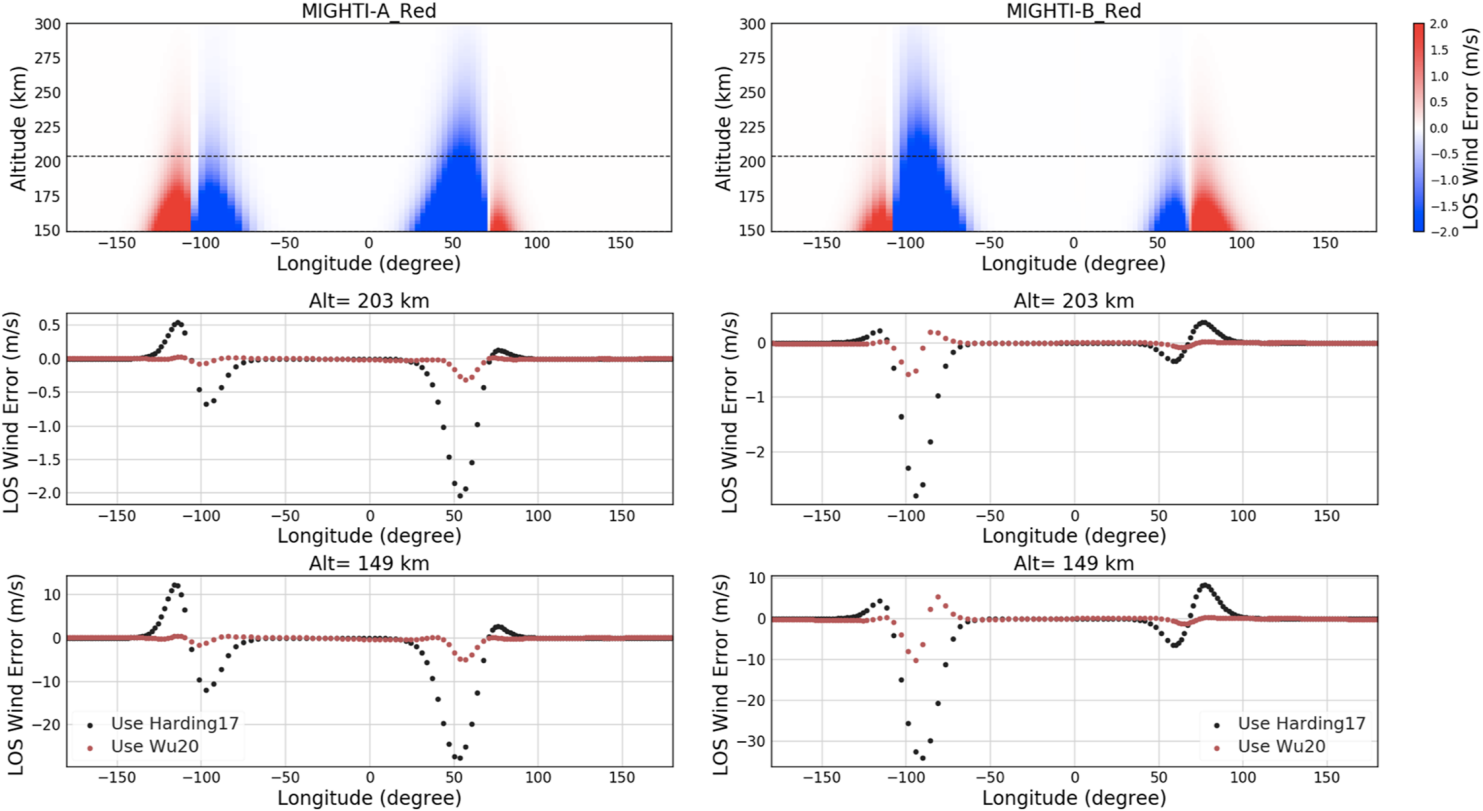
The retrieved MIGHTI‐A and MIGHTI‐B red line LOS wind error using symmetric airglow assumption (Harding17) and the correction using asymmetric airglow procedure (Wu20). (Top) The LOS wind error in all altitude levels for a completed orbit using Harding17. The dashed lines indicate the boundary altitudes demonstrated below. (Middle) The comparison between the retrieved LOS wind from Harding17 and Wu20 at 203 km, the lowest altitude for which both daytime and nighttime data in the red line are available in this case (1 January 2020). The black dotted line is for Harding17 whereas the red dotted line is for Wu20. (Bottom) Same as the middle panels but for the bottom altitude of the instrument FOV.

A forward model and an inversion algorithm are performed to understand the errors in the retrieved LOS wind due to the large VER gradient near the terminators. Figure 6 shows the error of the LOS wind retrieval of MIGHTI‐A and MIGHTI‐B from the red line emission. The top panel shows the LOS wind error using Harding17 at all altitudes for an entire orbit. The largest wind errors in the Harding17 run are as expected near the terminators, which is due to the asymmetric VER. The LOS wind is overestimated (LOS wind error >0) near the dayside parts of the terminators (−130° to −100° and 70° to 100°), whereas the LOS wind is underestimated (LOS wind error <0) near the nightside parts of the terminators (−100° to −70° and 30° to 70°).

The input wind profile (3) for the range of red line altitudes (149 to 300 km, in this case) decreases with increasing altitude and is negative at high altitudes. The overestimated and underestimated regions in Figure 6 (red and blue areas, respectively) are due to the fact that the contribution of the negative wind at the higher‐altitude layers in the onion peeling process used in Harding17 contaminates the wind retrieval at lower altitudes. The asymmetry in the shape of the underestimated and overestimated regions of the LOS wind is a function of viewing geometry with respect to the terminator boundary.

The middle and bottom panels in Figure 6 show the comparison between the retrieved LOS wind from Harding17 and Wu20 at particular altitudes. The middle plot in Figure 6 shows the errors at 203 km, which is the lowest altitude that nighttime data are expected to have sufficient signal‐to‐noise ratio for retrieving wind vectors. The largest error in the simulation using Harding17 on MIGHTI‐A (MIGHTI‐B) is −2.2 (−2.8) m/s, whereas the correction using Wu20 reduces the error by about a factor of 10 for both instruments in general, with a couple of exception data points around 50° and −100 °.

The bottom panels show the same comparison but for the 149 km altitude, which is the lowest altitude for daytime data. Due to the error propagation in the onion peeling process, it is not surprising that the error becomes larger at lower altitudes. The largest error in the simulation using Harding17 for MIGHTI‐A (MIGHTI‐B) is −28 m/s (−36 m/s), whereas the correction using Wu20 reduces the error by about a factor of 10 in general, and a factor of 4 at the longitudes where the effect of asymmetric VER is most significant. The result shows again that the asymmetric airglow procedure considered in Wu20 successfully improves the accuracy of the wind retrievals.

Since 99% of the data in the region of 200–300 km from the simulation using Harding17 have an error less than 10% of the mission uncertainty requirement of 8.7 m/s (Immel et al., 2018), the negative impact of the symmetric airglow assumption to the wind retrieval product above 203 km altitude is minor. That said, the impact of the symmetric airglow assumption to the wind retrievals in the lower altitudes close to the terminators is nonnegligible. Within 30° longitude from the terminators, the LOS wind error can be greater than the mission requirement in the altitude range 105–200 km in the daytime (Immel et al., 2018). Thus, the proposed algorithm will be important for studies using low‐altitude wind data near the terminator (e.g., studies of the ionospheric PRE)

**Figure 7:**
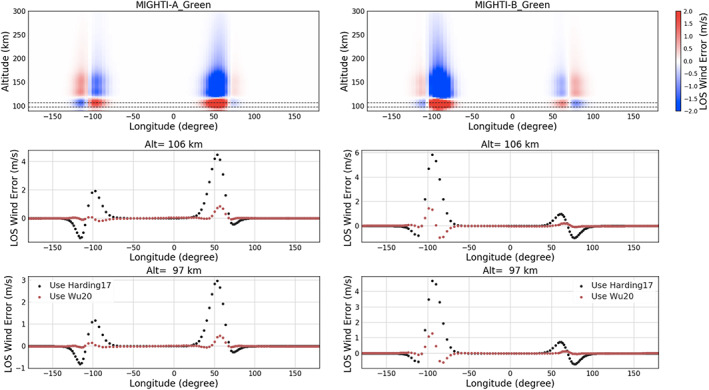
The retrieved MIGHTI‐A and MIGHTI‐B green line LOS wind error using symmetric airglow assumption (Harding17) and the correction using asymmetric airglow procedure (Wu20). (Top) The same as in Figure 6 (top) but for the green line case; (middle) the same as in Figure 6 (middle) but for 106 km, the highest altitude for which both daytime and nighttime data for the green line are available; (bottom) the same as the middle panels but for an altitude of 97 km, which is the closest altitude to the bottom layer of TIEGCM.

Similar to Figure 6, the retrieval cases for the green line are shown in Figure 7. The wind error in the wind retrievals from Harding17 in the top panel exhibits the effect of the asymmetric VER that was already discussed for the red line case. Since the same normalized longitudinal variation of VER (4) is applied for both green and red line cases, the most significant errors are found around the same longitude ranges as the red line case at both dawn and dusk terminators.

The middle panels in the example demonstrated in Figure 7 (106 km) is selected because it is the highest altitude for which the signal‐to‐noise ratio of both daytime and nighttime data for the green line are high enough to retrieve winds. The largest error in MIGHTI‐A (MIGHTI‐B) from the simulation using Harding17 is 4.3 m/s (6 m/s). The altitude of 97 km for the lower panels is the closest altitude to the bottom boundary of the TIEGCM2.0 (Maute, 2017), which is one of the models that is used to support interpretation of ICON observations. One of the applications of the ICON/MIGHTI wind product is used to derive the lower boundary forcing for TIEGCM by the Hough Mode Extension method (Maute, 2017). The largest error present in MIGHTI‐A (MIGHTI‐B) from the simulation using Harding17 is 4 m/s (5 m/s). Overall, similar to the result of the red line shown in Figure 6, the correction using Wu20 reduces the error by a factor of 10 for both instruments, and a factor of 3 at the longitudes where the effect of asymmetric VER is most significant.

Compared with the mission requirement of 8.7 m/s, the impact of the symmetric assumption on the green line product is minor. However, the error can be propagated and enhanced when more noise is introduced in the real data. Additionally, unlike random errors, the error in the onion peeling retrievals is not expected to reduce upon averaging.

## 4 The Trade‐Off Between Accuracy and Precision in Wu20

In section 3, we have simulated the LOS wind error for an entire simulated ICON orbit by using Harding17 and Wu20. Overall, the asymmetric airglow procedure in Wu20 effectively reduced the original error in Harding17 by about a factor of 10. However, as will be shown, the disadvantage of the Wu20 algorithm is its increased sensitivity to noise, so that the error propagation leads to worse precision.

We performed a Monte Carlo simulation to show the trade‐off between accuracy and precision at 70° latitude where Harding17 has the largest error for the MIGHTI‐A red line. We added noise to each row of a synthetic ICON interferogram to simulate measurement noise sources such as shot, dark, and read noise and then obtained the LOS wind errors from the retrieval of the simulations using Harding17 and Wu20. In total, 500 profiles with random noise are included in the analysis.

**Figure 8:**
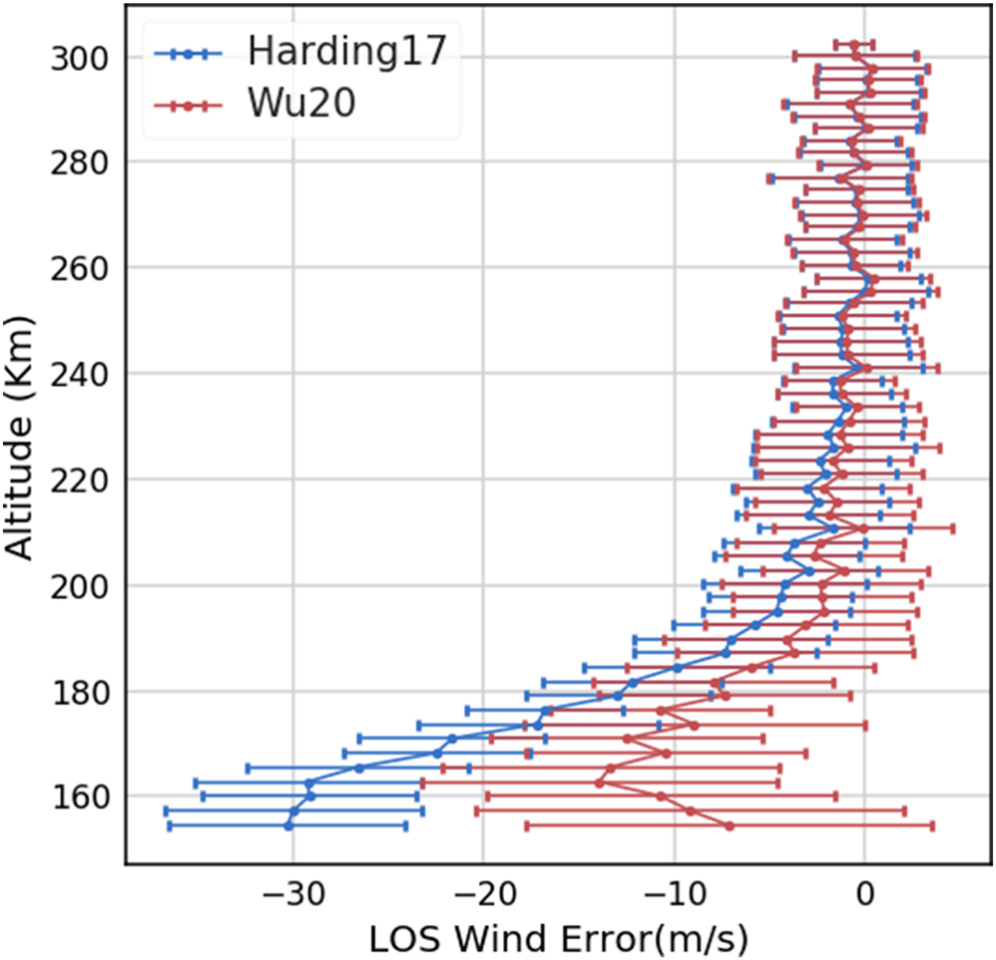
The analysis showing the trade‐off between precision and accuracy. The red profile is the LOS wind retrieval error using the Wu20 algorithm, and the blue profile is from Harding17. The error bar for each altitude levels marks one standard deviation of the error and represents the precision, while the line represents the accuracy.

In Figure 8, the red and blue profiles are the results using Wu20 and Harding17, respectively. The error bar at each altitude level marks one standard deviation of the LOS wind error obtained over 500 trials. The error bars of Wu20 are similar to Harding17 error bars at the very top of the profile but gradually increase at lower altitudes. This is the effect of the error propagation and enhancement from the onion peeling procedure. The result below 200 km shows that the mean LOS wind error is smaller in Wu20 than in Harding17, but the standard deviation (*σ*) is about two times larger than Harding17. In other words, it clearly shows that Wu20 improves the accuracy but loses 75% precision (1/*σ*^2^) compared to Harding17.

Although the trade‐off between accuracy and precision might limit the degree to which Wu20 can improve over Harding17, if the transition of the wind vector at the terminators is the target, Wu20 can still make significant correction for the lower altitudes and for the regions and periods with significant spatial airglow changes.

## 5 Conclusions

In this study, we described an algorithm to improve the accuracy of the wind vector retrieved from a spaceborne Doppler interfermetry instrument by considering the asymmetric VER before and after the tangent point of the FOV. The key step in the proposed algorithm is allowing the VERs to be different before and after the tangent point in the onion peeling procedure. A model run showed that if the VER variation along the LOS is well known, the input wind profile can be reconstructed to a higher accuracy using the asymmetric airglow procedure in Wu20 compared to Harding17.

An instrument that measures the Dopper shift in an interferogram to remotely retrieve the wind phase receives the integrated VER over the LOS; therefore, the VER variation along the LOS is generally unknown. One can reconstruct the VER variation by imaging tomography from different LOSs, but that requires viewing the same airglow from multiple directions, which is usually hard to implement by a single spacecraft. A simplified method with the assumption that the VER variation as a function of longitude is the same for all altitudes is demonstrated in this paper for a spaceborne measurement in a low‐inclination angle orbit like that of the ICON spacecraft. In this way, the continuous VER observation by MIGHTI itself provides the reference VER variation that can be applied in Wu20 and the actual MIGHTI wind retrievals.

We compared the accuracy of the LOS wind retrieval for a completed ICON orbit between Harding17 and Wu20. For the altitudes at which the signal‐to‐noise ratio of the emission is sufficient for retrieving LOS wind, the greatest impact of the symmetric airglow assumption of Harding17 to the ICON vector wind product is where the angle between the FOV and the terminator boundary is the greatest within a 30° longitude range from the terminators, which causes an error of at least 10 m/s. Our results showed that Wu20 can effectively reduce this error by a factor of 10 in general. Although Wu20 improves the accuracy, it loses precision by 75% compared to Harding17. Note that these results only apply to the simulations conducted in this study and could vary depending on circumstances such as different shape of VER variation in longitude and VER changes with season and solar flux.

A few caveats are worth noting when applying Wu20 to the other Abel transform‐related retrieval algorithms. The algorithm in Wu20 assumes that VER can change along LOS the in addition to changing in altitude, while the wind only changes in altitude. This limitation in uniform wind assumption has not been relaxed in Wu20. In the demonstration of the application on ICON/MIGHTI, we assumed that the VER ratio (*R*_*mn*,*ξ*_) only varies with longitude; however, the VER variability in altitude is also worth examining in future studies. Furthermore, VER variations caused by sources other than the solar terminator will also be considered in future studies.

## Data Availability

The ICON data can be found online (at https://icon.ssl.berkeley.edu/Data).
